# Altered Expression of Somatostatin Receptors in Pancreatic Islets from NOD Mice Cultured at Different Glucose Concentrations *In Vitro* and in Islets Transplanted to Diabetic NOD Mice *In Vivo*

**DOI:** 10.1155/2011/623472

**Published:** 2011-09-05

**Authors:** Eva Ludvigsen, Mats Stridsberg, Eva T. Janson, Stellan Sandler

**Affiliations:** ^1^Department of Medical Cell Biology, Uppsala University, Biomedicum, P.O. Box 571, 75123 Uppsala, Sweden; ^2^Section of Clinical Chemistry, Department of Medical Sciences, University Hospital, Uppsala University, 75185 Uppsala, Sweden; ^3^Section of Endocrine Oncology, Department of Medical Sciences, University Hospital, Uppsala University, 75185 Uppsala, Sweden

## Abstract

Somatostatin acts via five receptors (sst_1–5_). We investigated if the changes in pancreatic islet sst expression in diabetic NOD mice compared to normoglycemic mice are a consequence of hyperglycemia or the ongoing immune reaction in the pancreas. Pancreatic islets were isolated from NOD mice precultured for 5 days and further cultured for 3 days at high or low glucose before examined. Islets were also isolated from NOD mice and transplanted to normal or diabetic mice in a number not sufficient to cure hyperglycemia. After three days, the transplants were removed and stained for sst_1–5_ and islet hormones. Overall, changes in sst islet cell expression were more common in islets cultured in high glucose concentration *in vitro* as compared to the islet transplantation *in vivo* to diabetic mice. The beta and PP cells exhibited more frequent changes in sst expression, while the alpha and delta cells were relatively unaffected by the high glucose condition. Our findings suggest that the glucose level may alter sst expressed in islets cells; however, immune mechanisms may counteract such changes in islet sst expression.

## 1. Introduction

Somatostatin is a peptide hormone, known to have several biological functions, and its effects are mediated via five specific membrane-bound receptors (sst_1-5_). In rodents, somatostatin inhibits secretion of several hormones such as insulin, glucagon, GH, TSH, and prolactin. It can also regulate gastrointestinal motility, gastric acid, and exocrine pancreatic secretion [1–5]. Immunohistochemical studies have shown that all five ssts are expressed on the pancreatic islets of rodents and that the intensity and distribution of ssts varies between the subtypes, the islet cell type, and species [6–8].

Type 1 diabetes mellitus is considered to be caused by autoimmune destruction of the pancreatic beta cells, rendering the pancreas unable to synthesize and secrete sufficient amounts of insulin. The nonobese diabetic (NOD) mouse is an animal model with many features resembling human type 1 diabetes, such as infiltrating immune cells (insulitis) accompanied by spontaneous hyperglycaemia [9]. We have shown that diabetic NOD mice have a higher sst expression in the pancreatic islets as compared to normoglycemic NOD mice [1]. The mechanism behind this receptor upregulation in diabetic mice is not clear, but it may be either a consequence of hyperglycemia or due to immune reactions in the NOD mouse. To address this issue, we have performed both *in vivo* syngeneic islet transplantations to diabetic as well as normoglycemic NOD mice and cultured NOD mouse islets *in vitro* at different glucose concentrations and subsequently evaluated islet sst expression. The rationale for this design is that the *in vitro* system would provide an environment with only an elevated glucose concentration, whilst *in vivo* after islet transplantation both an influence from autoimmunity and a diabetic milieu can be envisaged.

## 2. Materials and Methods

### 2.1. Animals, Islet Isolation, and Culture

Inbred NOD mice (Biomedical Center, Uppsala, Sweden), originally obtained from the Clea Company, Aobadi, Japan, were used for this study. The animals had free access to water and pelleted food and were housed in a room with a 12-hour light/dark cycle. All experiments were approved by the local animal ethics committee at Uppsala University (Uppsala, Sweden) and were in accordance with international guidelines (NIH publications 85–23). Blood glucose determinations (Medisense, Waltham, MA, USA) were performed on blood samples taken from the tail tip, and the weight of the animal was measured. 

For islet isolation, male NOD mice (4–7 weeks of age) were killed by cervical dislocation followed by resection of the pancreas. Pancreatic islets were isolated by collagenase digestion procedure [10], and islets were hand picked with a braking pipette under stereomicroscope. Islets were precultured 5–7 days in 4.5 mL RPMI 1640 medium (Sigma-Aldrich, St. Louis, Mo, USA) containing 11.1 mM glucose, supplemented with 10% fetal calf serum (FCS, vol/vol), benzylpenicillin (100 U/mL), and streptomycin (0.1 mg/mL), in an atmosphere of humidified air +5% CO_2_ before experiments. Medium was changed every second day.

To investigate the effect of glucose on sst expression *in vitro*, groups of 100 pancreatic islets (*n* = 4) were cultured in RPMI 1640 + 10% FCS supplemented with 5.6 mM or 28.8 mM glucose for three days (e.g., [11]). The medium was changed on the second day.

### 2.2. Islet Transplantation beneath the Kidney Capsule

A female NOD mouse was considered diabetic when the nonfasting blood glucose concentration exceeded 10 mM and served as recipient of grafted islets to diabetic mice, whereas females with blood glucose below 10 mM were designated as normoglycemic. Normoglycemic (*n* = 6) and diabetic female (*n* = 6) NOD mice were anaesthetised with an intraperitoneal injection of 0.02 mL/g body weight of Avertin (a 2.5% solution of 10 g 97% 2,2,2-tribromoethanol (Sigma-Aldrich) in 10 mL 2-methyl-2-butanol (Kemila, Stockholm, Sweden)). Cultured pancreatic islets, 200 islets per graft, were collected in a braking pipette and implanted beneath the left kidney capsule. It should be noted that the number of implanted islets was on purpose chosen to be too low to normalize the hyperglycemia of the diabetic mice. The blood glucose levels were determined daily, and the animals were killed after three days, and the graft-bearing kidney was dissected free. The part of the kidney containing the islet graft was excised leaving a margin of 1 mm of each side of the graft.

### 2.3. Staining with sst_1–5_ Antibodies

The production and specificity of subtype-specific somatostatin receptor antibodies has been described earlier [7, 12]. The grafts or cultured islets were fixed in 10% buffered formalin (Merck, Darmstadt, Germany) for 24 hours at room temperature and then changed to 70% ethanol and embedded in paraffin. Sections, 5 *μ*m thick, were cut and attached to Polysine glass slides (Menzel-Gläser, Braunschweig, Germany).

The specimens were single stained for the five sst receptors [7]. The immune reaction was amplified by an avidin-biotin complex coupled to alkaline phosphates (Vectastain ABC-AP, Vector Laboratories Inc, Burlingame, Calif, USA) and visualized with Vector Red (Vector Laboratories), as substrate [7]. When the ssts-specific antibodies were preincubated with the peptides used for immunization, the immunoreactivity for each receptor was blocked (data not shown), [7]. Moreover, an extensive description of the generation, peptide specificity, and other characteristics of the sst antibodies is given in [7].

### 2.4. Coexpression of sst_1–5_ and Islet Cells

To investigate the coexpression of sst_1-5_ on the transplanted islet cells as well as the cultured islets, we used the immunofluorescence method described previously [7]. The transplants from normoglycemic female NOD mice and diabetic female NOD mice or islets cultured in different glucose concentrations were collected, paraffin embedded, and stained for sst_1-5_ in a cocktail with chicken anti-insulin (1 : 750, Immunsystem, Uppsala, Sweden), chicken antiglucagon (1 : 400, a kind gift from Professor Anders Larsson, Uppsala University, Uppsala, Sweden; raised against human glucagon (Novo Nordisk, Bagsvaerd, Denmark)), sheep antisomatostatin (1 : 25, Guildhay, Guildford, UK), or sheep anti-PP (1 : 25, SeroTech, Oxford, UK) as previously described [7]. The immune reaction was visualized by a cocktail consisting of secondary antibodies Cy3-conjugated donkey antirabbit IgG (1 : 100, Jackson ImmunoResearch, West Grove, Pa) and Cy2-conjugated donkey antisheep IgG (1 : 100, Jackson ImmunoResearch) or Cy2-conjugated donkey antichicken IgG (1 : 100, Jackson ImmunoResearch). The specificity of the commercially employed antihormone antibodies was tested by using different dilutions of the antibodies as well as omitting the primary antibodies. Concerning the generation, peptide specificities, and characterization of antibodies against the different sst-receptors, an in-depth description can be found in [7]. 

### 2.5. Morphological Evaluation

In the double fluorescence study, the kidney grafts or cultured islets were examined in a Leica Leitz DMR fluorescence microscope (Leica Microsystems, Wetzlar, Germany) equipped with filters of 492 nm to 510 nm for Cy2 (green) and 550 nm to 570 nm for Cy3 (red). Pictures from a Zeiss Axiocam camera (Carl Zeiss, Oberkochen, Germany) of each transplants, using both filters, were merged together with Adobe Photoshop 7.0 software (Adobe, San Jose, Calif, USA), in which a yellow colour indicated coexpression of the sst subtype with any of the four islet hormones tested in this study. The results are expressed as a percentage of ssts-positive cells in relation to the total number of the respective islet cell type in a specific pancreatic islet.

### 2.6. Statistical Analysis

Data are presented as means ± SEM, and groups of data were compared using Student's *t*-test, where *P* < 0.05 was considered as statistically significant. The computer program used was SigmaStat 2.0 (SPSS Science, Chicago, Ill, USA).

## 3. Results

### 3.1. Animals

**Figure 1:**
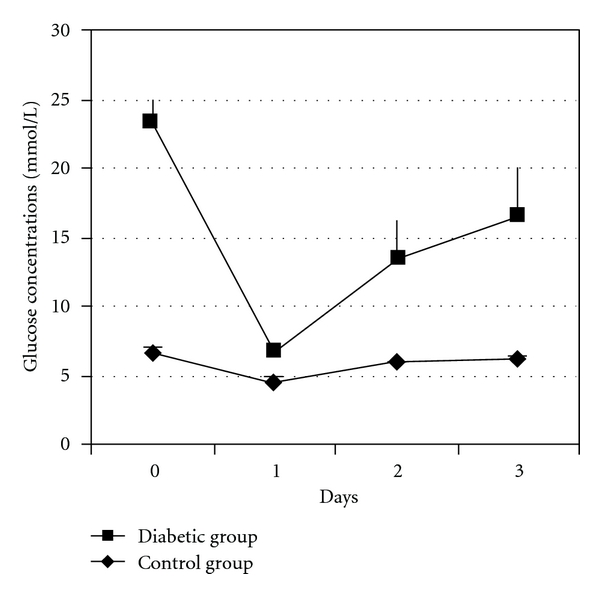
Blood glucose levels were determined every day for three days in the transplanted NOD mice in the diabetic (*n* = 6) and control (*n* = 6) groups.

The blood glucose concentrations for the transplanted mice during three days are shown in Figure 1. The value on day 0 was obtained immediately before the operation and the day 3 value when the graft was removed. As can be seen in the figure, the implanted number of islets was not sufficient to cure the hyperglycemia, thus the mice remained diabetic, and the implanted islets were exposed to a diabetic state *in vivo* at least during the last two days. NOD mouse islets grafted into normoglycemic female NOD mice were used as control, and these mice remained normoglycemic throughout.

### 3.2. Coexpression of sst_1–5_ with Islets Hormones

To investigate the influence of elevated glucose concentrations on the islet sst expression pattern, grafts or cultured islets were double stained with sst_1-5_ antibodies together with the islet hormones insulin, glucagon, somatostatin, and PP. 

**Figure 2:**
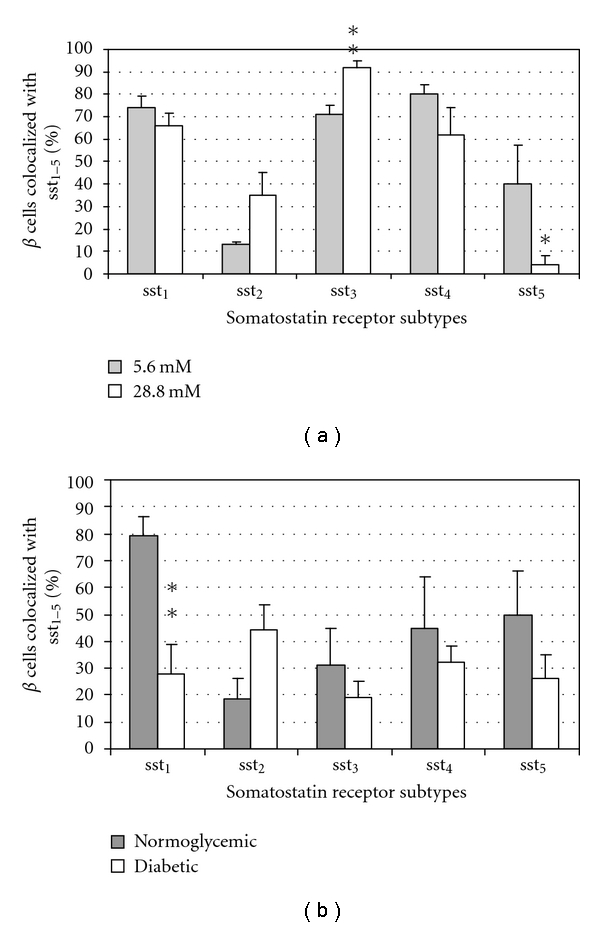
Quantitative analysis of the immunohistochemical expression, using a double-labelling procedure, of ssts in beta cells in isolated pancreatic islets cultured in different glucose concentrations ((a), *n* = 4) or in the transplanted islets grafts ((b), *n* = 6). Bars represent the means ± SEM from the percentage of cells positive for the given ssts. *P < 0.05, **P < 0.01 versus control using Student's unpaired *t*-test.

In Figure 2, the quantitative analysis of the coexpression of sst_1-5_ with insulin, that is, beta cells in *in vitro* cultured islets (Figure 2(a)) or transplanted islets (Figure 2(b) is shown. The sst_3_ expression in beta-cells was significantly upregulated in islets cultured at 28.8 mM glucose, while the sst_5_ expression was significantly decreased as compared to islets cultured at 5.6 mM. No significant differences in expression were observed for sst_1_, sst_2_, and sst_4_. In the *in vivo* setting, the expression of all ssts remained unchanged except for sst_1_ which was significantly reduced in the islets removed from diabetic animals.

**Figure 3:**
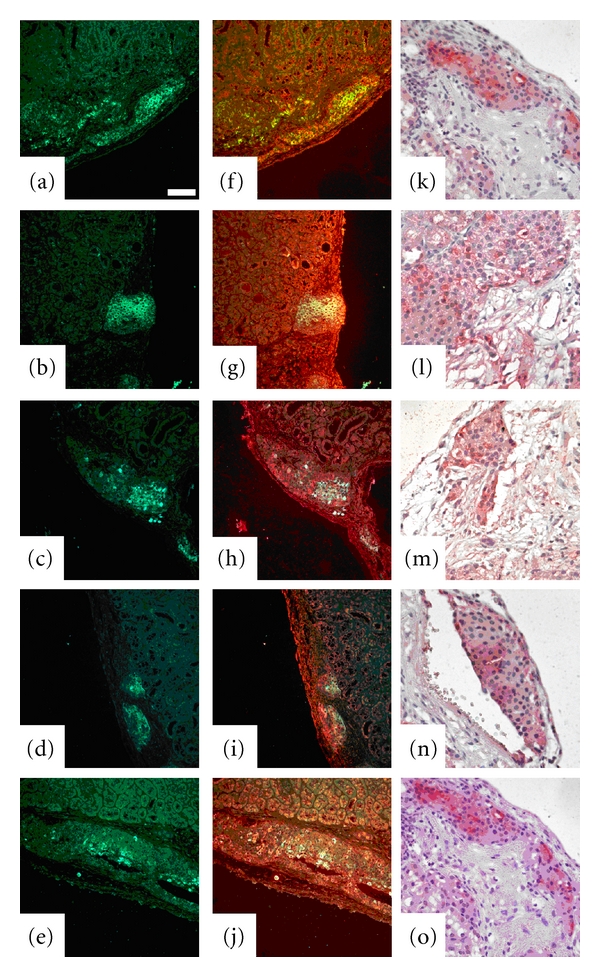
Syngeneic pancreatic islets in groups of 200 islets were transplanted beneath the kidney capsule, and the animal was sacrificed on day 3 after transplantation. A representative graft in a transplanted diabetic NOD mouse stained for insulin (green, (a)–(e)) coexpressing sst_1-5_ (yellow, (f)–(j)). (k)–(o) show a representative graft in transplanted diabetic NOD mice single stained with sst_1-5_-specific antibodies. Bar = 50 *μ*m.

Figure 3 shows the distribution of insulin-positive cells (green, Figures 3(a)–3(e)) and the merged picture of cells coexpressing sst_1-5_ (yellow, Figures 3(f)–3(j)) in a representative section of a transplanted pancreatic islet from a diabetic NOD mouse. Sections of transplanted NOD mouse islets under the kidney capsule single stained for sst_1-5_ are shown in Figures 3(k)–3(o). The grafts were harvested already on day 3 with the intent that the process of so-called recurrence of disease with immune cell infiltration should be minimal [12, 13]. The kidney tissue was also weakly stained by the ssts antibodies suggesting that renal tissue also expresses sst.

**Figure 4:**
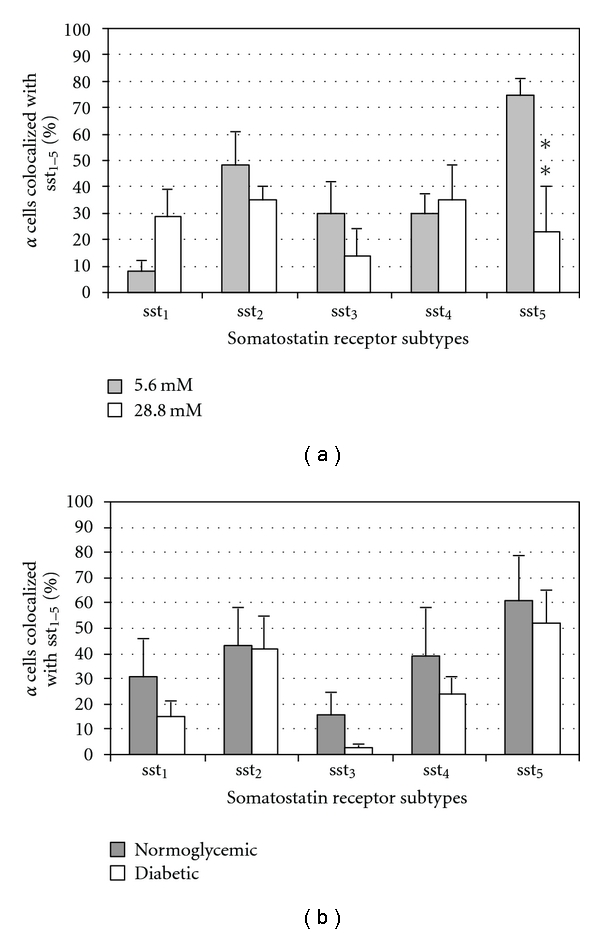
Quantitative analysis of the immunohistochemical expression, using a double-labelling procedure, of ssts in alpha cells in isolated pancreatic islets cultured in different glucose concentrations ((a), *n* = 4) or in the transplanted islets grafts ((b), *n* = 6). Bars represent the means ± SEM from the percentage of cells positive for the given ssts. *P < 0.05, **P < 0.01 versus control using Student's unpaired *t*-test.

There was no significant difference in sst_1_–sst_4_ expression on alpha cells between islets cultured at 5.6 mM and 28.8 mM glucose, while sst_5_ was significantly decreased when islets were cultured at high glucose levels (Figure 4(a)). All ssts remained unchanged on alpha cells from transplanted islets (Figure 4(b)). 

**Figure 5:**
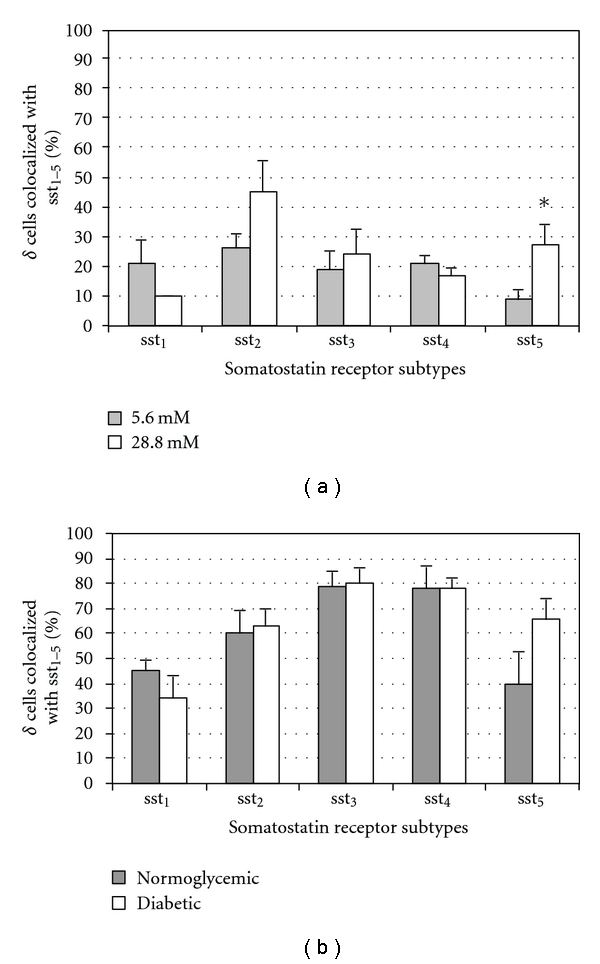
Quantitative analysis of the immunohistochemical expression, using a double-labelling procedure, of ssts in delta-cells in isolated pancreatic islets cultured in different glucose concentrations ((a), *n* = 4) or in the transplanted islets grafts ((b), *n* = 6). Bars represent the means ± SEM from the percentage of cells positive for the given ssts. *P < 0.05, **P < 0.01 versus control using Student's unpaired *t*-test.

Overall, the sst expression on delta cells in cultured islets was fairly low (Figure 5(a)). However, the sst_5_ expression was significantly increased at the high glucose concentration. The overall sst expression on delta cells in transplanted islets was higher, but equal when comparing transplants from normoglycemic and diabetic animals (Figure 5(b)).

**Figure 6:**
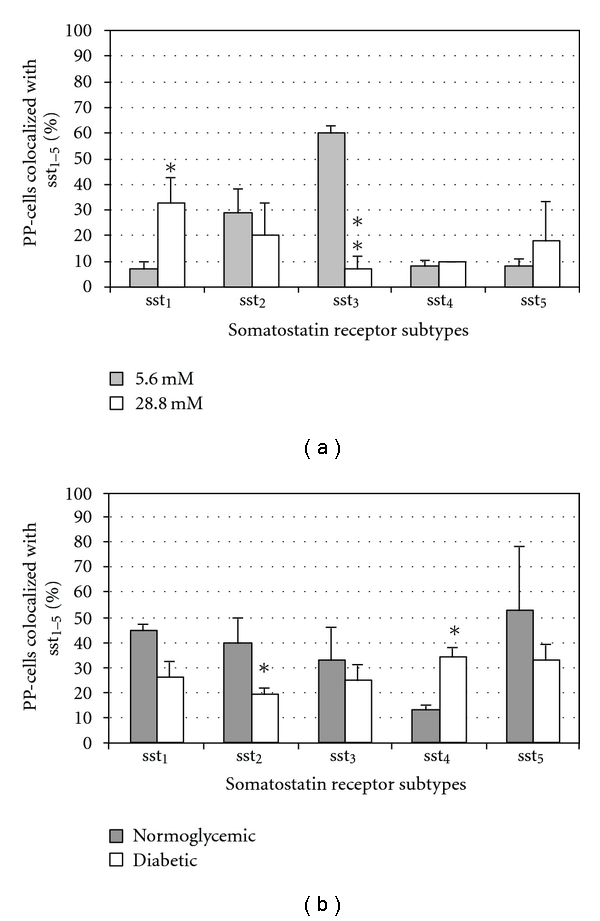
Quantitative analysis of the immunohistochemical expression, using a double-labelling procedure, of ssts in PP cells in isolated pancreatic islets cultured in different glucose concentrations ((a), *n* = 4) or in the transplanted islets grafts ((b), *n* = 6). Bars represent the means ± SEM from the percentage of cells positive for the given ssts. *P < 0.05, **P < 0.01 versus control using Student's unpaired *t*-test.

On PP cells in islets cultured at 28.8 mM glucose, an increase of sst_1_ and a decrease in sst_3_ expression was observed (Figure 6(a)), while the expression of the other sst remained unchanged. In transplanted islets, PP cells showed a decrease in sst_2_ and an increase in sst_4_ expression, while the other ssts remained unchanged (Figure 6(b)).

## 4. Discussion

We have previously shown that all sst subtypes are expressed in rodent pancreatic islets, but on the expression varies between the four major islet cell types [7]. Furthermore, this sst expression was increased in diabetic compared to normoglycemic NOD mice, particularly for sst_2_–sst_5_ [1]. The reason for this latter finding was not clear and the aim of the present study was to investigate if the increased ssts expression observed in pancreatic islets of diabetic NOD mice depends on an elevated glucose concentration *in vivo* or merely reflects a response to the ongoing immune reaction in the pancreas of the NOD mice. 

NOD mouse pancreatic islets were cultured *in vitro* for three days in media containing either 5.6 mM glucose or 28.8 mM glucose to elucidate the effect of an elevated glucose concentration without a putative influence of immune-related factors present *in vivo*, for example, cytokines. In parallel precultured islets isolated from young normoglycemic NOD mice were transplanted under the kidney capsule of diabetic NOD mice. A suboptimal number of islets were implanted, which means that the diabetic animals remained hyperglycaemic, and the grafted islets were exposed to a prolonged period of high glucose *in vivo*. The grafts were removed after three days, before any marked signs of graft destruction by invading immune cells following so-called recurrence of disease [13, 14]. If a later time point after transplantation had been selected, it is anticipated that the evolving graft destruction and further increasing blood glucose concentrations would become confounding factors when evaluating the current data. 

The changes in sst expression observed after either culture at high glucose or after islet transplantation to diabetic recipients are schematically summarized in Table 1. More alterations in islet cell expression of ssts were induced in the *in vitro* setting compared to those observed *in vivo* following islet transplantation. It cannot be excluded that the *in vitro* environment allows more glucose-induced changes in sst expression to become manifest than *in vivo*, since compensatory extraislet mechanisms conveyed by nerves and blood flow may counteract such changes. In addition, the ambient experimental glucose concentration (28.8 mM) *in vitro* was higher in comparison to the glucose concentration of the transplanted diabetic mice (15–20 mM). It is possible that this difference in islet exposure to glucose had an impact on changes in islet cell sst expression. Moreover, the changes observed *in vitro* did not correspond to changes obtained *in vivo*, which could argue in favour of the assumption that the level of glucose is of importance. 

The combined analysis of the *in vivo* and *in vitro* data suggests that the alpha and delta cells were relatively unaffected by the high-glucose conditions, whilst beta and PP cells exhibited more dynamic changes. Concerning the specific sst, it was found that *in vitro* exposure to high glucose affected sst_5_ expression on alpha, beta, and delta cells. Sst_5_ activation is suggested to inhibit the insulin secretion in rodents [8, 15–17]. Higher insulin levels are needed to meet the demand of an elevated glucose concentration. It may therefore be suggested that the decrease in sst_5_ expression seen in this study is a downregulation of receptor expression to avoid signalling which may inhibit the insulin secretion from beta cells. Interestingly a similar decrease in beta cell sst_5_ expression was found in the spontaneously diabetic NOD mice [1]. The effect of the increased sst_5_ expression on delta cells is unclear, but it might contribute to an autocrine-mediated dampered secretion of somatostatin which would promote insulin secretion. 

The expression of sst_3_ was increased on beta cells in isolated islets cultured at higher glucose concentrations. The insulin-producing cell line MIN6 has previously been reported to express sst_3_, and this was suggested to be related to MAP kinase and c-fos signalling in inhibition of cell growth by somatostatin [18]. An upregulation of sst_3_ by a high glucose concentration could result in a restriction on beta-cell proliferation promoted in a milieu of a high-functional demand on the beta cells. Moreover, sst_3_ may also affect signal transduction associated with adenyl cyclase activity [2], which could be of relevance for beta-cell function since cAMP is an important amplifier of insulin secretion [19].

PP secretion is inhibited by somatostatin and by elevated blood glucose levels [20]. At higher concentrations of glucose *in vitro*, the sst_1_ expression was increased and sst_3_ expression decreased on PP cells. It is possible that these combined changes in expression of sst is part of the regulation of the PP-cell response to high glucose. Further studies, however, are needed to address this question.

## 5. Conclusion

In our earlier study in NOD mice, we reported a higher sst expression in the pancreatic islets of diabetic mice as compared to normoglycemic NOD mice [1]. The mechanism behind this upregulation in diabetic mice was unclear, but it may be either a consequence of hyperglycemia or due to immune reactions in the NOD mouse. Our data from this study indicates that hyperglycemia alone is not enough to explain the changes observed in NOD mice *in vivo*. Either immune-related mechanisms or other *in vivo* regulatory mechanisms may counteract effects of elevated glucose on islet cell sst expression. Future studies should address the mechanisms of the immune response for the changes in sst expression in pancreatic islets observed in NOD mice with a spontaneously developed diabetes.

## Figures and Tables

**Table 1:** Summary of changes in islet cell sst expression in islets cultured at high glucose and in islet transplanted to hyperglycemic NOD mice.

	Cultured islets				Transplanted islets			
	28 mM glucose versus 5.6 mM glucose				Diabetic versus normoglycemic			
Cell	*α*	*β*	*δ*	PP	*α*	*β*	*δ*	PP
sst_1_	→	→	→	↑	→	↓	→	→
sst_2_	→	→	→	→	→	→	→	↓
sst_3_	→	↑	→	↓	→	→	→	→
sst_4_	→	→	→	→	→	→	→	↑
sst_5_	↓	↓	↑	→	→	→	→	→

→ denotes no significant change, ↓ denotes a decline in the fraction of cells expressing the sst, and ↑ denotes an increase in the fraction of cells expressing the sst.
